# Engineering Placental Mesenchymal Stem Cells with PEDF for Retinal Protection in Diabetic Retinopathy

**DOI:** 10.3390/antiox15040473

**Published:** 2026-04-10

**Authors:** Jaeyeon Kim, Se Jin Hong, Jeong Woo Choi, Jin Seok, Youngje Sung, Gi Jin Kim

**Affiliations:** 1 Division of Life Sciences, Department of Life Science, Graduate School, CHA University, Seongnam-si 13488, Republic of Korea; janejykim92@gmail.com (J.K.); sejin1451@gmail.com (S.J.H.); why12525@chauniv.ac.kr (J.W.C.); 2 PLABiologics Co., Ltd., Seongnam-si 13522, Republic of Korea; jseok@plabiologics.com; 3 Department of Ophthalmology, CHA Bundang Medical Center, CHA University, Seongnam-si 13488, Republic of Korea

**Keywords:** diabetic retinopathy, stem cell therapy, placenta-derived mesenchymal stem cells

## Abstract

Diabetic retinopathy (DR) is a major cause of adult blindness and is characterized by progressive retinal vascular dysfunction and pathological angiogenesis. To establish a DR model, streptozotocin (STZ) was intraperitoneally injected into rats. After 8 weeks, naïve placenta-derived mesenchymal stem cells (PD-MSCs) or PEDF-overexpressing PD-MSCs (PD-MSCs^PEDF^) were intravitreally transplanted into the right eye for 4 weeks. Pathological neovascularization in DR is regulated by the balance between vascular endothelial growth factor (VEGF) and pigment epithelium-derived factor (PEDF). In diabetic retinas, increased VEGF and decreased PEDF expression were reversed following PD-MSC transplantation. Notably, PD-MSCs^PEDF^ treatment resulted in higher PEDF, and lower VEGF expression compared with naïve PD-MSCs, with similar expression patterns observed in the contralateral non-transplanted eyes. These findings indicate that engineering PD-MSCs^PEDF^ enhances anti-angiogenic activity by modulating VEGF and PEDF balance, thereby alleviating vascular damage in STZ-induced diabetic retinas.

## 1. Introduction

Diabetic retinopathy (DR), a common complication of diabetes mellitus, is one of the leading causes of adult blindness in developed countries [1]. DR is characterized by dysfunction of the retinal vascular system, primarily driven by increased vascular permeability resulting from anatomical and biochemical alterations in the retina [2]. However, recent studies have redefined DR as a complex neurovascular disorder in which neuronal dysfunction and glial activation occur early in disease progression, often preceding overt vascular abnormalities. Hyperglycemia-induced metabolic stress disrupts neuronal signaling and synaptic integrity, leading to progressive neuronal loss. Concurrently, Müller glial cells undergo reactive gliosis, characterized by impaired metabolic support, dysregulated glutamate clearance, and secretion of pro-inflammatory mediators. These neuroglial alterations exacerbate inflammatory responses, disrupt retinal homeostasis, and contribute to both neurodegeneration and secondary vascular dysfunction [3]. Accumulating evidence indicates that oxidative stress plays a critical role in the development and progression of DR. Persistent hyperglycemia enhances mitochondrial production of reactive oxygen species (ROS) and disrupts the antioxidant defense system, leading to activation of cell-death pathways and inflammatory responses. These oxidative insults ultimately cause structural and functional retinal damage through the denaturation of DNA, proteins, and lipids. Consequently, targeting oxidative stress has emerged as an important therapeutic strategy for the prevention and management of DR [4].

Current treatments for advanced DR include laser photocoagulation, intravitreal injections of anti-vascular endothelial growth factor (VEGF) agents, and glucocorticoid therapy [5]. Anti-VEGF therapy is widely used to control vascular abnormalities primarily mediated by VEGF activity; however, its therapeutic efficacy remains limited due to the multifactorial and complex pathophysiology of diabetes. Given that VEGF inhibition remains essential for regulating pathological angiogenesis and vascular leakage in DR, otherwise, the overexpression of pigment epithelium-derived factor (PEDF), which confers retinal protective effects alongside VEGF suppression, can be considered as a complementary therapeutic approach. This approach can suppress oxidative stress and VEGF expression, thereby reducing vascular permeability, preventing pathological neovascularization, and inhibiting platelet activation through the regulation of NADPH oxidase activity [6]. Vascular stabilization by antioxidants may promote photoreceptor survival and ultimately confer protective effects in the retina.

Recently, mesenchymal stem cell (MSC)-based therapies have gained attention as promising treatments for retinal degenerative diseases, including DR [7]. Among various MSC sources, human placenta-derived mesenchymal stem cells (PD-MSCs) possess several advantages, such as strong immunomodulatory and anti-inflammatory properties, the ability to home to injury sites, ease of isolation, high proliferative capacity, and minimal ethical concerns [8]. Moreover, MSCs secrete a wide range of paracrine factors that create a protective microenvironment in the diabetic retina by enhancing neuroprotective signaling and reducing oxidative stress [9]. Supporting this mechanism, MSC administration in a streptozotocin-induced diabetic nephropathy model attenuated apoptosis, ER stress, and inflammation while improving tissue histopathology, suggesting that MSCs primarily act by modulating diabetes-associated cellular stress rather than through direct tissue replacement [10]. Transplanting stem cells has the advantage of less immunity or damage. Various treatments are being developed for diabetic retinopathy using adult mesenchymal stem cells [11,12]. Several in vitro and in vivo studies suggest that MSC-based therapy represents a viable therapeutic approach for DR [13]. Notably, intravitreal administration of umbilical cord-derived MSCs has been shown to confer neuroprotection and modulate pathological processes in streptozotocin (STZ)-induced diabetic mouse models [14]. To further enhance therapeutic efficacy, genetic modification of MSCs has been explored to enable overexpression of specific factors involved in retinal protection and regeneration [15]. In our previous studies, we developed genetically modified PD-MSCs overexpressing PEDF using a nonviral gene delivery system. PEDF-overexpressing PD-MSCs (PD-MSCs^PEDF^) improved mitochondrial function through antioxidant activity and activated survival signaling pathways associated with retinal regeneration in both H_2_O_2_-injured rat models and retinal pigment epithelial cells in vitro [16]. However, their therapeutic feasibility in diabetic retinopathy is still unclear.

Therefore, the present study aimed to compare the therapeutic effects of naïve PD-MSCs and PD-MSCs^PEDF^ in diabetic retinopathy, to evaluate their antioxidant and vascular function effects through modulation of the VEGF/PEDF balance, and to elucidate their therapeutic mechanisms in STZ-induced diabetic rats.

## 2. Materials and Methods

### 2.1. Cell Culture

The human retinal pigment epithelium-derived cell line ARPE-19 (ATCC, Manassas, VA, USA) was maintained in DMEM/F-12 (Dulbecco’s Modified Eagle Medium/Nutrient Mixture F-12; Gibco, Carlsbad, CA, USA) supplemented with 10% fetal bovine serum (FBS) (Gibco) and 1% penicillin/streptomycin (P/S) (Gibco). Placenta-derived mesenchymal stem cells (PD-MSCs) (IRB-07-18) and PEDF-overexpressing PD-MSCs (PD-MSCs^PEDF^) were cultured in MEM α GlutaMAX™ (MEM α, GlutaMAX™ Supplement, no nucleosides; Gibco, Carlsbad, CA, USA) containing 10% FBS, 25 ng/mL human fibroblast growth factor-4 (FGF4) (PeproTech, Rocky Hill, NJ, USA), 1 μg/mL heparin (Sigma-Aldrich, St. Louis, MO, USA), and 1% P/S. All cells were maintained at 37 °C in a humidified atmosphere containing 5% CO_2_.

### 2.2. In Vitro Model

Human retinal pigment epithelial cells (ARPE-19) were seeded at a density of 1 × 10^5^ cells per well in 6-well plates and cultured for 24 h. Cells were maintained in DMEM/F-12 supplemented with 10% FBS under standard culture conditions and used at approximately 70–80% confluency. ARPE-19 cells between passage 6–15 were used for all in vitro experiments. The culture medium was then replaced with DMEM/F-12 medium supplemented with an additional 25 mM glucose (Sigma-Aldrich, St. Louis, MO, USA), resulting in a final glucose concentration of approximately 42.5 mM to establish high-glucose conditions. After 24 h of glucose treatment, the medium was removed. The in vitro model was established as previously described [17]. For the co-culture experiments, Transwell inserts with an 8-μm pore size (Falcon, Marlboro, NY, USA) were placed into each well. Naïve PD-MSCs or PEDF-overexpressing PD-MSCs (PD-MSCs^PEDF^) were seeded into the inserts at a density of 5 × 10^4^ cells per insert and co-cultured with ARPE-19 cells for an additional 24 h prior to sample collection. Following co-culture, the inserts were removed, and the ARPE-19 cells were collected for subsequent analyses.

### 2.3. Animal Model

Seven-week-old male Sprague–Dawley rats (KOATECK Inc., Pyeongtaek, Republic of Korea) were maintained in an air-conditioned animal facility under standard laboratory conditions. Diabetes was induced by a single intraperitoneal injection of streptozotocin (STZ) (60 mg/kg; Sigma-Aldrich), as previously described [18]. Rats with blood glucose levels below 250 mg/dL after STZ injection were excluded from the study. The remaining animals were randomly assigned to the following experimental groups: Sham control (Con) (n = 6), STZ-induced non-transplantation (NTx), (n = 6), Naïve PD-MSCs transplantation (PD-MSCs) (n = 12), PEDF-overexpressing PD-MSCs transplantation (PD-MSCs^PEDF^) (n = 11). At 8 weeks after STZ injection, rats in the transplantation groups received an intravitreal injection of naïve PD-MSCs or PD-MSCs^PEDF^ (2 × 10^5^ cells/eye). Animals were sacrificed 4 weeks after transplantation for subsequent analyses. All experimental procedures were approved by the Institutional Animal Care and Use Committee (IACUC) of CHA University, Seongnam, Republic of Korea (IACUC No. 250146).

### 2.4. Blood Chemistry

Serum biochemical parameters, including ALT (alanine aminotransferase), AST (aspartate aminotransferase), albumin, total cholesterol, triglycerides (TG), high-density lipoprotein (HDL), low-density lipoprotein (LDL), blood urea nitrogen (BUN), creatinine, and the BUN/creatinine ratio, were analyzed in serum samples from STZ-induced rats by DooYeol Biotech Institute (Seoul, Republic of Korea).

### 2.5. Quantitative Real-Time Polymerase Chain Reaction (qRT-PCR)

Total RNA was extracted from liver tissues using TRIzol reagent (Invitrogen, Carlsbad, CA, USA), followed by chloroform (Sigma-Aldrich, St. Louis, MO, USA) and isopropanol (Merck, Darmstadt, Germany) extraction according to the manufacturer’s instructions. The concentration and purity of RNA were determined using a spectrophotometer. Complementary DNA (cDNA) was synthesized from 1 µg of total RNA using SuperScript III reverse transcriptase and RNaseOUT (Invitrogen, Carlsbad, CA, USA). Quantitative real-time PCR was performed using gene-specific primers and SYBR Green Master Mix (Roche, Basel, Switzerland) on a real-time PCR system under the following conditions: initial denaturation at 95 °C for 10 min, followed by 40 cycles of 95 °C for 15 s and 55–60 °C for 1 min. Relative gene expression levels were calculated using the 2^−ΔΔCt^ method. Rat GAPDH was used as an internal control for normalization. All experiments were performed in triplicate. Primer sequences and their corresponding melting temperatures are listed in Tables S1–S3.

### 2.6. Enzyme-Linked Immunosorbent Assay (ELISA)

The concentrations of insulin, C-peptide, and HbA1c in rat serum were measured using commercial ELISA kits (Abcam, Cambridge, UK) according to the manufacturer’s instructions. Serum samples were collected and diluted as required prior to analysis. Standard curves were generated for each assay using the provided standards, and concentrations were calculated accordingly. Optical density (OD) was measured at 450 nm using an Epoch microplate reader (BioTek, Winooski, VT, USA). All experiments were performed in duplicate, and the mean values were used for statistical analysis.

### 2.7. Histopathological Analysis

Eyeballs were fixed in 10% formalin and methanol for 24 h, embedded in paraffin, and sectioned at a thickness of 5 μm. Sections were obtained from the central region of the eyeball at the level of the optic disk and subjected to hematoxylin and eosin (H&E) staining. Stained sections were scanned using a digital histological scanner (3DHISTECH Ltd., Budapest, Hungary). Histological analysis was performed using digital images, and representative sections were selected for presentation. All experiments were performed in triplicate.

### 2.8. Immunofluorescence Staining

For analysis of the localization of angiogenic and anti-angiogenic markers, immunofluorescence staining was performed. Briefly, tissue sections were permeabilized with 0.1% Triton X-100 in PBS and blocked with Protein block serum-free (Dako, Carpinteria, CA, USA) for 1 h at room temperature to prevent nonspecific binding. The sections were then incubated overnight at 4 °C with primary antibodies against vascular endothelial growth factor (VEGF; 1:200; Novus Biologicals, CO, USA) and pigment epithelium-derived factor (PEDF; 1:200; LSbio, Washington, DC, USA). After washing with PBS, sections were incubated with the secondary antibody, Alexa Fluor 488-conjugated anti-rabbit IgG (Invitrogen, Carlsbad, CA, USA), for 1 h at room temperature. Nuclei were counterstained with 4′,6-diamidino-2-phenylindole (DAPI) (Invitrogen, Carlsbad, CA, USA). Images were acquired using a confocal laser scanning microscope (Zeiss, Oberkochen, Germany). All experiments were performed in triplicate.

### 2.9. Statistical Analysis

All data were analyzed using GraphPad Prism version 9.0 (GraphPad Software, San Diego, CA, USA). Statistical comparisons were performed using Student’s *t*-test or one-way analysis of variance (ANOVA), as appropriate. A *p* value < 0.05 was considered statistically significant.

## 3. Results

### 3.1. PD-MSCs^PEDF^ Transplantation Improves Retinal Cellular Integrity in STZ-Induced Diabetic Rats

**Figure 1:**
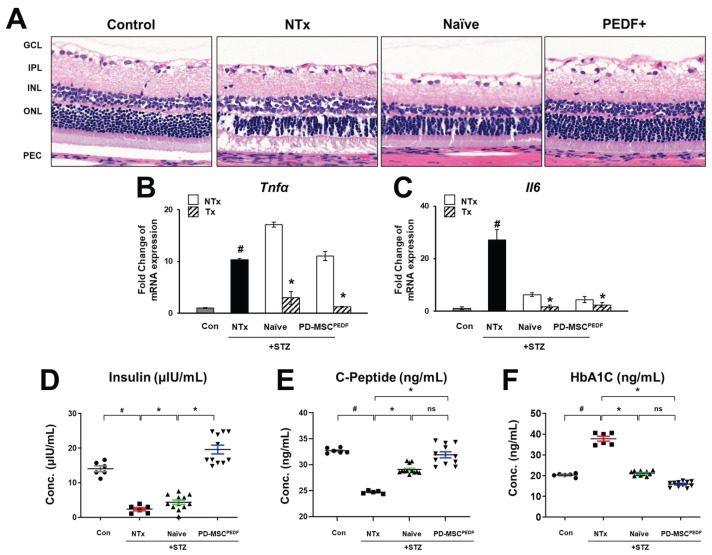
PD-MSCs^PEDF^ transplantation improves retinal cellular integrity in STZ-induced diabetic rats. (**A**) Histological evaluation of retinal morphology in STZ-induced rat eyeballs by hematoxylin and eosin (H&E) staining. Images were acquired using a 20× objective. mRNA expression levels of the pro-inflammatory cytokines (**B**) *Tnfa* and (**C**) *Il6* in STZ-induced rat retinas. # vs. Con group; * vs. NTx group. Serum levels of (**D**) insulin, (**E**) C-peptide, and (**F**) HbA1c in STZ-induced rats. Data are presented for the following experimental groups: Control (Con), STZ-induced non-transplantation (NTx), STZ-induced intravitreal transplantation of naïve PD-MSCs (Tx Naïve), and STZ-induced intravitreal transplantation of PEDF-overexpressing PD-MSCs (Tx PEDF+). *p* < 0.05. # Con vs. NTx; * NTx vs. Tx; * Tx Naive vs. Tx PEDF+; ns; not significant.

To establish a rat model of diabetic retinopathy rat model, streptozotocin (STZ; 60 mg/kg) was intraperitoneally administered, whereas the sham control group received balanced salt solution (BSS) only (Control). 8 weeks after induction, naïve placenta-derived mesenchymal stem cells (PD-MSCs; Naïve) or PEDF-overexpressing PD-MSCs (PD-MSCs^PEDF^) were intravitreally transplanted into the right eye and retinal outcomes were evaluated 4 weeks later. Animals were randomly divided into four groups: Control (n = 6), STZ-induced MSC non-transplanted group (NTx; n = 6), naïve PD-MSC transplantation group (Naïve; n = 12), and PD-MSCs^PEDF^ transplantation group (PEDF+; n = 11). Histological analysis revealed marked retinal degeneration in STZ-induced rats. Compared with the Control group, the NTX group exhibited substantial structural disruption in both the inner nuclear layer (INL) and outer nuclear layer (ONL). Transplantation of naïve PD-MSCs partially attenuated these changes, whereas PEDF-overexpressing PD-MSCs preserved retinal structure more effectively (Figure 1A). We next evaluated retinal inflammation. Expression of pro-inflammatory cytokines tumor necrosis factor-alpha (*Tnfa*) and interleukin-6 (*Il6*) was significantly elevated in NTx group relative to controls. In contrast, both transplantation groups showed markedly reduced expression, with no significant difference between the Naïve and PEDF+ groups (Figure 1B,C). Systemic metabolic parameters were also examined. STZ-treated rats displayed decreased insulin and C-peptide levels together with elevated HbA1c. These metabolic abnormalities were partially restored by PD-MSC transplantation and were more effectively improved in the PEDF+ group than in the Naïve group (Figure 1D–F). Collectively, these findings indicate that PEDF-overexpressing PD-MSC transplantation was associated with preservation of retinal architecture and amelioration of systemic metabolic abnormalities in STZ-induced diabetic rats.

### 3.2. PD-MSCs^PEDF^ Transplantation Enhances Retinal-Specific Gene Expression in an STZ-Induced Diabetic Rat Model

**Figure 2:**
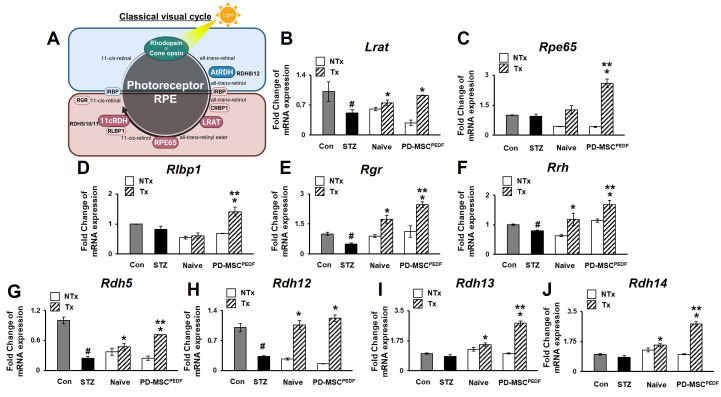
PD-MSCs^PEDF^ transplantation enhances retinal-specific gene expression in an STZ-induced diabetic rat model. (**A**) Schematic illustration of the visual cycle between photoreceptors and the retinal pigment epithelium (RPE). (**B**–**J**) Relative mRNA expression levels of retinal-specific and visual cycle-related genes. Data are shown for the following experimental groups: Control, STZ (STZ-induced diabetic rats), Naïve (STZ + intravitreal transplantation of naïve PD-MSCs), and PEDF+ (STZ + intravitreal transplantation of PD-MSCs^PEDF^). *p* < 0.05. # vs. Con group; * vs. NTx group; ** vs. Naive group.

Diabetic retinopathy is characterized by impaired retinal function and disruption of the visual cycle [19]. To assess whether PD-MSC transplantation affects visual cycle-related gene expression in STZ-induced diabetic retinopathy (Figure 2A), retinal pigment epithelium (RPE) and photoreceptor-associated genes were analyzed by quantitative real-time PCR (qRT-PCR). Expression of RPE-associated visual cycle genes, including lecithin retinol acyltransferase (*Lrat*), retinal pigment epithelium-specific protein 65 (*Rpe65*), retinaldehyde binding protein 1 (*Rlbp1*), retinal G protein-coupled receptor (*Rgr*), retinal pigment epithelium-derived rhodopsin homolog (*Rrh*), and retinol dehydrogenase 5 (*Rdh5*), was significantly reduced in the NTx group compared with the Control group. Transplantation of PD-MSCs increased the expression of these genes, and higher expression levels were observed in the PEDF-overexpressing PD-MSC group than in the naïve PD-MSC group (Figure 2B–G). Given the functional coupling between the RPE and photoreceptors in the visual cycle [20], we next examined photoreceptor-associated genes. Expression of retinol dehydrogenase 12 (*Rdh12*), retinol dehydrogenase 13 (*Rdh13*), and retinol dehydrogenase 14 (*Rdh14*) was significantly enhanced in the PEDF+ group compared with the Naïve group (Figure 2H–J). In addition, RPE65, a key enzyme required for the regeneration of the visual chromophore 11-cis-retinal in the RPE, exhibited markedly increased expression following transplantation of PD-MSCs^PEDF^, indicating enhanced restoration of the visual cycle machinery.

### 3.3. PD-MSCs^PEDF^ Transplantation Supports Mitochondrial Homeostasis and Antioxidant Capacity in the Retina of STZ-Induced Diabetic Rats

**Figure 3:**
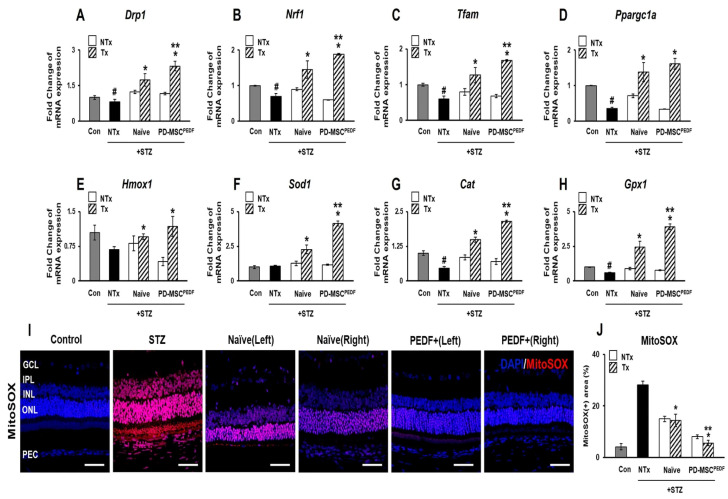
PD-MSCs^PEDF^ transplantation supports mitochondrial homeostasis and antioxidant capacity in the retina of STZ-induced diabetic rats. Relative mRNA expression levels of mitochondrial biogenesis and antioxidant markers: (**A**) *Drp1*, (**B**) *Nrf1*, (**C**) *Tfam*, (**D**) *Ppargc1a*, (**E**) *Hmox1*, (**F**) *Sod1*, (**G**) *Cat*, and (**H**) *Gpx1*. Localization and intensity of mitochondrial reactive oxygen species (ROS) detected by MitoSOX staining are shown by immunofluorescence (**I**) and quantified in (**J**). Scale bar = 100 µm. Data are presented for the following experimental groups: Control, STZ (STZ-induced diabetic rats), Naïve (STZ + intravitreal transplantation of naïve PD-MSCs), and PEDF+ (STZ + intravitreal transplantation of PD-MSCs^PEDF^). *p* < 0.05. # vs. Con group; * vs. NTx group; ** vs. Naive group.

Pigment epithelium-derived factor (PEDF) has been reported to stabilize mitochondrial function and protect retinal pigment epithelium (RPE) cells against oxidative stress [21]. In our previous study, we demonstrated that PEDF-overexpressing PD-MSCs enhanced mitochondrial biogenesis and antioxidant capacity in oxidative stress-induced RPE cells in vitro [22]. To further assess whether PD-MSCs^PEDF^ transplantation affects mitochondrial status in STZ-induced diabetic rat retinas, we analyzed the expression of genes associated with mitochondrial biogenesis and antioxidant defense. Expression of mitochondrial biogenesis-related genes, including dynamin-related protein 1 (*Drp1*), nuclear respiratory factor 1 (*Nrf1*), mitochondrial transcription factor A (*Tfam*), and peroxisome proliferator-activated receptor gamma coactivator 1-alpha (*Ppargc1a*), was significantly higher in the PEDF+ group compared with the Naïve group (Figure 3A–D). A similar pattern was observed for antioxidant enzyme genes. Expression of heme oxygenase 1 (*Hmox1*), superoxide dismutase 1 (*Sod1*), catalase (*Cat*), and glutathione peroxidase 1 (*Gpx1*) was elevated in the PEDF+ group (Figure 3E–H). As diabetes-associated oxidative stress is known to impair mitochondrial function by reducing antioxidant capacity and increasing reactive oxygen species (ROS) production, we next assessed mitochondrial ROS using MitoSOX staining. Consistent with the gene expression data, mitochondrial ROS signal intensity in retinal sections was reduced in the PEDF-overexpressing PD-MSC group relative to the naïve PD-MSC group (Figure 3I,J). Collectively, these results indicate that PD-MSCs^PEDF^ transplantation is accompanied by coordinated changes in mitochondrial and antioxidant gene expression together with reduced mitochondrial ROS accumulation in STZ-induced diabetic rat retinas.

### 3.4. PD-MSCs^PEDF^ Transplantation Modulates Angiogenic and Anti-Angiogenic Factor Expression in STZ-Induced Diabetic Rat Retinas

**Figure 4:**
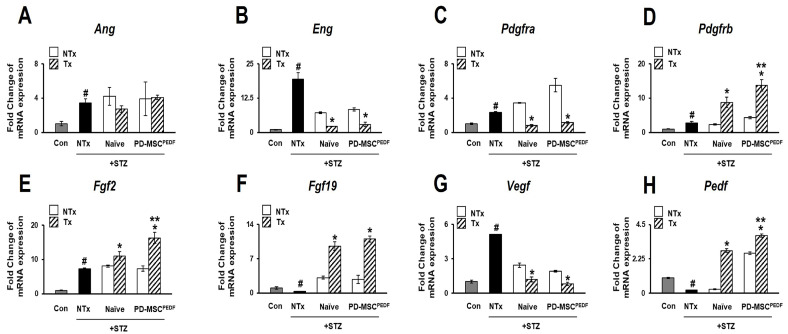
Changes in angiogenesis-related gene expression following transplantation of PD-MSCs^PEDF^ into diabetic rat retinas. Relative mRNA expression levels of angiogenesis-related factors in STZ-induced rat eyeballs analyzed by quantitative real-time PCR (qRT-PCR): (**A**) *Ang*, (**B**) *Eng*, (**C**) *Pdgfra*, (**D**) *Pdgfrb*, (**E**) *Fgf2*, (**F**) *Fgf19*, (**G**) *Vegf*, and (**H**) *Pedf*. Data are presented for the following experimental groups: Control, STZ (STZ-induced diabetic rats), Naïve (STZ + intravitreal transplantation of naïve PD-MSCs), and PEDF+ (STZ + intravitreal transplantation of PD-MSCs^PEDF^). *p* < 0.05. # vs. Con group; * vs. NTx group; ** vs. Naive group.

Pathological neovascularization contributes to the progression of diabetic retinopathy and subsequent visual impairment [23]. To examine the impact of PD-MSC transplantation on angiogenesis in diabetic retinas, we quantified expression of angiogenesis-related genes in streptozotocin (STZ)-induced rats. Expression of angiogenin (*Ang*), endoglin (*Eng*), and platelet-derived growth factor receptor-α (*Pdgfra*) was elevated in the NTx group compared with both transplantation groups. In contrast, expression of platelet-derived growth factor receptor-β (*Pdgfrb*), basic fibroblast growth factor 2 (*Fgf2*), and fibroblast growth factor 19 (*Fgf19*), which was reduced in the NTx group, increased following PD-MSC transplantation (Figure 4A–F). We next assessed expression of vascular endothelial growth factor (*Vegf*) and pigment epithelium-derived factor (*Pedf*). In the NTx group, Vegf expression was increased, whereas Pedf expression was decreased relative to controls (Figure 4G). Compared with the naïve PD-MSC group, transplantation of PEDF-overexpressing PD-MSCs was associated with lower *Vegf* expression together with higher *Pedf* expression (Figure 4H). Together, these data show that PD-MSC transplantation alters angiogenesis-related gene expression in STZ-induced diabetic retinas, with PEDF-overexpressing PD-MSCs displaying a greater shift in the VEGF–PEDF expression profile.

### 3.5. PD-MSCs^PEDF^ Protect RPE Cells from High Glucose-Induced Damage Through Elevated Antioxidants and Reduced VEGF Expression

**Figure 5:**
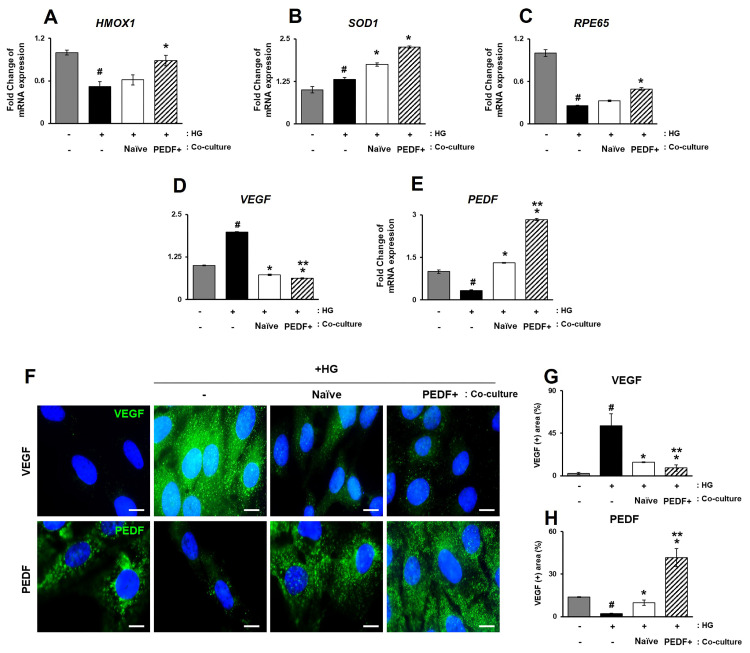
PD-MSCs^PEDF^ protect RPE cells from high glucose-induced damage through elevated antioxidants and reduced VEGF expression. Relative mRNA expression levels of antioxidant and retinal function-related genes in high glucose-treated ARPE-19 cells analyzed by quantitative real-time PCR (qRT-PCR; (**A**) *HMOX1*, (**B**) *SOD1*, and (**C**) *RPE65*). Relative mRNA expression levels of *VEGF* and *PEDF* are shown in (**D**,**E**), respectively. Immunofluorescence analysis showing the localization of VEGF and PEDF is presented in (**F**–**H**). Scale bar = 10 µm. Data are shown for the following experimental groups: Control (Con), HG (high glucose-treated cells), Naïve (high glucose + co-culture with naïve PD-MSCs), and PEDF+ (high glucose + co-culture with PD-MSCs^PEDF^). *p* < 0.05. # vs. Con group; * vs. HG group; ** vs. Naive group.

**Figure 6:**
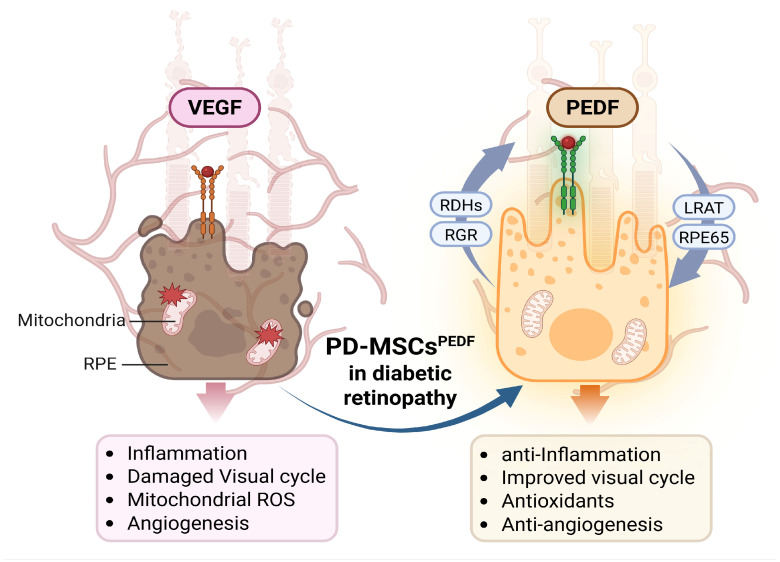
Schematic model illustrating PEDF overexpressing PD-MSCs restore RPE homeostasis and visual cycle function while suppressing inflammation, mitochondrial oxidative stress, and pathological angiogenesis in diabetic retinopathy. This figure was Created in BioRender. Kim, J. (2026) https://BioRender.com/4cvbge9 (accessed on 7 April 2026).

RPE cells play a critical role in the protection and functional maintenance of photoreceptors [24]. Hyperglycemia-induced oxidative stress is a major contributor to RPE injury in diabetic retinopathy. To assess the effects of PEDF-overexpressing PD-MSCs on high glucose (HG)-induced RPE injury, human ARPE-19 cells were exposed to high glucose conditions (25 mM) as an in vitro DR model and subsequently co-cultured with naïve PD-MSCs or PD-MSCs^PEDF^. Following co-culture, the expression of antioxidant enzymes, RPE-specific markers, and angiogenesis-related factors were evaluated. As shown in Figure 5A–C, the mRNA expression levels of antioxidant enzymes, including *HMOX1* and *SOD1*, which were reduced under HG conditions, were significantly increased following co-culture with PD-MSCs^PEDF^ (Figure 5A,B). In addition, the expression level of the RPE-specific marker RPE65 was also elevated in the PD-MSCs^PEDF^ co-culture condition (Figure 5C). Given that increased and reduced PEDF expression are associated with pathological retinal neovascularization [25], the expression levels of VEGF, and PEDF were further examined. qRT-PCR revealed that VEGF expression levels were significantly increased in HG-treated RPE cells compared with control cells, whereas PEDF expression was decreased (Figure 5D,E). In contrast, compared with co-culture with naïve PD-MSCs, PD-MSCs^PEDF^ co-culture was associated with reduced VEGF expression and increased PEDF expression at the mRNA levels. Immunofluorescence analysis demonstrated that VEGF and PEDF were predominantly localized in the cytoplasm of RPE cells. Consistent with gene expression data, HG-treated RPE cells exhibited increased VEGF and decreased PEDF signals. Notably, HG-treated RPE cells co-cultured with PD-MSCs^PEDF^ showed inverse expression patterns of VEGF and PEDF compared with those co-cultured with naïve PD-MSCs (Figure 5F–H). Collectively, these results indicate that PD-MSCs^PEDF^ co-culture is associated with enhanced antioxidant responses and modulation of VEGF/PEDF-related signaling in high glucose-induced RPE cells (Figure 6).

## 4. Discussion

Glucose homeostasis is tightly regulated in the body, primarily through hepatic glucose metabolism involving glycogen synthesis and breakdown. However, chronic hyperglycemia leads to excessive glucose accumulation in the bloodstream, which promotes systemic inflammation and oxidative stress [26]. These pathological conditions affect not only metabolic organs such as the liver and pancreas but also intracellular organelles, including mitochondria. Accumulating evidence indicates that elevated glucose levels increase reactive oxygen species (ROS) production, resulting in mitochondrial DNA damage and mitochondrial dysfunction [27]. Because mitochondria play an essential role in retinal metabolism and the visual cycle, mitochondrial dysfunction in retinal tissue can contribute to impaired retinal function and the progression of retinal diseases, including diabetic retinopathy (DR) [28].

Consistent with these observations, our STZ-induced diabetic rat model exhibited hyperglycemia accompanied by reduced insulin expression and increased mitochondrial ROS levels, confirming the establishment of a diabetic and oxidative stress-associated retinal environment (Figure 1 and Figure 3). These findings support the relevance of mitochondrial oxidative stress as a key pathological component in DR. In addition, chronic hyperglycemia promotes the accumulation of advanced glycation end products (AGEs), which further exacerbate endothelial dysfunction through activation of AGE–RAGE signaling pathways [29]. This process enhances inflammatory responses, increases vascular permeability, and disrupts nitric oxide bioavailability, ultimately contributing to diabetic microangiopathy. Moreover, oxidative stress-induced endothelial damage stimulates aberrant angiogenic signaling, including VEGF upregulation, thereby promoting pathological neovascularization in diabetic retinopathy [30].

Current therapeutic strategies for DR, such as laser photocoagulation, intravitreal anti-VEGF injections, and vitreoretinal surgery, are primarily aimed at delaying disease progression rather than restoring retinal function. As an alternative approach, mesenchymal stem cell (MSC)-based therapies have gained attention due to their paracrine, immunomodulatory, anti-oxidative, and cytoprotective properties through regulation of apoptosis-related pathways [31]. Previous studies have demonstrated that intravitreal transplantation of adult MSCs can preserve retinal vasculature and attenuate retinal damage in diabetic and ischemic retinal models [32]. In line with these findings, our results indicate that transplantation of placenta-derived MSCs (PD-MSCs), particularly PEDF-overexpressing PD-MSCs (PD-MSCs^PEDF^), was associated with improved retinal structural integrity, restoration of retinal functional markers, and modulation of visual cycle-related gene expression (Figure 1 and Figure 2).

Angiogenic imbalance is a hallmark of DR, characterized by increased vascular endothelial growth factor (VEGF) expression and reduced levels of pigment epithelium-derived factor (PEDF), which together promote pathological neovascularization [33]. Notably, VEGF and PEDF function as a counter-regulatory axis in the diabetic retina, in which hypoxia-induced HIF-1α activation elevates VEGF while concomitantly suppressing PEDF expression, thereby shifting the angiogenic balance toward neovascularization [34]. Consistently, restoration of this balance has been shown to alleviate retinal injury, as modulation of the HIF-1α/VEGF pathway accompanied by increased PEDF expression reduced vascular leakage and retinal damage in experimental diabetic models. Importantly, PEDF itself acts as a functional anti-angiogenic effector, as increased PEDF derived from retinal pigment epithelium directly suppresses endothelial cell proliferation, migration, and tube formation while promoting apoptosis [35]. Furthermore, hypoxic stress in retinal pigment epithelium reduces PEDF secretion, and anti-VEGF agents have also been shown to downregulate PEDF production, suggesting that current therapies may not fully restore the angiogenic homeostasis of the diabetic retina [36]. Accordingly, anti-VEGF therapies are widely used to control abnormal angiogenesis in DR. [36]. Consistent with these concepts, PD-MSC transplantation reduced VEGF expression while increasing PEDF expression in diabetic retinas, with PD-MSCs^PEDF^ producing a more pronounced normalization of the VEGF/PEDF balance than naïve PD-MSCs (Figure 4).

Beyond its anti-angiogenic activity, PEDF also functions as a critical regulator of cellular stress responses in retinal tissues. Previous studies have shown that PEDF attenuates oxidative stress-induced apoptosis in retinal pigment epithelium (RPE) cells through activation of PI3K/AKT signaling pathways [37]. Ultrastructural analyses further demonstrated that PEDF-overexpressing PD-MSCs improve mitochondrial morphology and integrity under oxidative stress conditions, including increased mitochondrial volume fraction and cristae density [16,38]. In addition, the imbalance in the VEGF-to-PEDF ratio is mechanistically linked to mitochondrial oxidative stress in diabetic retinopathy, where reduction in mitochondrial ROS normalizes VEGF and PEDF expression and improves retinal pathology [39]. Consistent with these reports, our findings indicate that PD-MSCs^PEDF^ transplantation is associated with enhanced expression of mitochondrial-related and antioxidant genes and reduced mitochondrial ROS levels in diabetic retinas, suggesting a supportive role for PEDF in maintaining mitochondrial homeostasis under diabetic stress.

Collectively, this study demonstrates that PEDF-overexpressing PD-MSCs exert beneficial effects in STZ-induced diabetic retinopathy by modulating oxidative stress, mitochondrial function, angiogenic balance, thereby improving retinal functional markers. Notably, these effects are closely associated with the restoration of endothelial homeostasis and attenuation of diabetes-induced microangiopathy and pathological neovascularization. While further studies are required to elucidate the precise molecular mechanisms and long-term therapeutic potential, these findings support the potential utility of PD-MSCs^PEDF^ as a complementary cell-based strategy for mitigating diabetic retinal damage. However, several limitations should be acknowledged. First, this study was conducted using an STZ-induced diabetic animal model, which predominantly reflects acute hyperglycemia-driven β-cell toxicity and may not fully recapitulate the complex and heterogeneous pathophysiology of human diabetic retinopathy. In particular, human DR involves chronic metabolic dysregulation, neurovascular degeneration, and sustained inflammatory responses that are not entirely reproduced in this model. Moreover, other experimental models—including genetic and ischemia-driven models—capture distinct aspects of disease progression, further highlighting the limitation of relying on a single model system. In addition, only male animals were used to reduce experimental variability, which may further limit the generalizability of our findings, as potential sex-specific differences were not evaluated. Importantly, our findings were not validated in human retinal tissues, and thus the translational relevance of PD-MSCs^PEDF^ in human DR remains to be established. Second, although we observed significant improvements in retinal structure, mitochondrial function, and angiogenic balance, the long-term efficacy and durability of PD-MSCs^PEDF^ transplantation were not assessed. In particular, the persistence of therapeutic effects and the potential need for repeated administration remain unclear. Furthermore, PD-MSCs^PEDF^ were administered via systemic intravenous injection in this study, whereas local intravitreal delivery is more commonly used in clinical settings for retinal diseases. Therefore, differences in delivery routes may influence cell distribution, retinal targeting efficiency, and therapeutic outcomes, which were not addressed in this study.

Third, while our data suggest that modulation of oxidative stress and the VEGF/PEDF axis plays a central role, the precise molecular mechanisms linking PEDF overexpression to mitochondrial regulation and endothelial cell function remain incompletely defined. Specifically, downstream signaling pathways and cell-type-specific interactions were not directly addressed in this study. In this context, ARPE-19 cells were used at 70–80% confluence to maintain experimental consistency; however, this condition may not fully reflect a differentiated, physiologically relevant phenotype observed at higher confluence. Therefore, the in vitro finding should be interpreted with this limitation in mind. Finally, translation of these findings into clinical applications will require validation in human tissues and well-controlled clinical studies.

## 5. Conclusions

In conclusion, intravitreal transplantation of PD-MSCs, including PEDF-overexpressing PD-MSCs (PD-MSCs^PEDF)^, improves retinal structural integrity and mitochondrial function in a diabetic retinopathy model. These beneficial effects are associated with the attenuation of oxidative stress and restoration of angiogenic balance, as evidenced by reduced VEGF expression and increased PEDF levels, thereby contributing to the preservation of retinal homeostasis. Notably, PD-MSCs^PEDF^ exhibited enhanced therapeutic efficacy compared with naïve PD-MSCs, suggesting that targeted modulation of the VEGF/PEDF axis represents a key mechanism underlying their protective effects. Collectively, these findings highlight the potential of PD-MSCs^PEDF^ as a promising cell-based therapeutic strategy for the treatment of diabetic retinopathy.

## Data Availability

The original contributions presented in this study are included in the article/Supplementary Material. Further inquiries can be directed to the corresponding author.

## Supplementary Materials

The following supporting information can be downloaded at https://www.mdpi.com/article/10.3390/antiox15040473/s1, Figure S1: Diabetic metabolism in STZ-induced rat model; Table S1: Rat primer sequences using qRT-PCR (1); Table S2: Rat primer sequences using qRT-PCR (2); Table S3: Human primer sequences using qRT-PCR.

## Abbreviations

The following abbreviations are used in this manuscript:

DR	Diabetic retinopathy
STZ	Streptozotocin
ROS	Reactive oxygen species
PEDF	Pigment epithelium-derived factor
VEGF	Vascular endothelial growth factor
PD-MSCs	Placenta-derived-mesenchymal stem cells
NTx	Non-transplantation
Tx	Transplantation
TNF-α	Tumor necrosis factor-α
IL-6	interleukin-6
LRAT	Lecithin retinol acyltransferase
RPE65	Retinal pigment epithelium-specific protein 65
RLBP1	retinaldehyde binding protein 1
RGR	Retinal G protein-coupled receptor
RRH	Retinal pigment epithelium-derived rhodopsin homolog
RDH5	Retinol dehydrogenase 5
RBP1	Retinol binding protein 1
RDH12	Retinol dehydrogenase 12
RDH13	retinol dehydrogenase 13
RDH14	retinol dehydrogenase 14
DRP1	Dynamin-related protein 1
NRF1	Nuclear respiratory factor 1
TFAM	Mitochondrial transcription factor A
PPARGC1A	Peroxisome proliferator-activated receptor gamma coactivator 1-alpha
HMOX1	Heme oxygenase 1
SOD1	Superoxide dismutase 1
CAT	Catalase
GPX1	Glutathione peroxidase 1
PDGFα	Platelet-derived growth factor receptor-α
PDGFRβ	Platelet-derived growth factor receptor-β
FGF2	basic Fibroblast growth factor
FGF19	Fibroblast growth factor 19
