# Supplementary material for: Engineering Placental Mesenchymal Stem Cells with PEDF for Retinal Protection in Diabetic Retinopathy
Source: Antioxidants (Basel). 2026 Apr 10;15(4):473. doi: 10.3390/antiox15040473 (PMC13113140; doi:10.3390/antiox15040473)

Table S1. Rat primer sequences using qRT-PCR (1)

Gene	Accession number	Sequence (5’ - 3’)	Tm (℃)
TNF-α	NM_012675.3	F: 5’-ATACACTGGCCCGAGGCAAC-3’<br>R: 5’-CCACATCTCGGATCATGCTTTC-3’	60
IL-6	NM_012589.2	F: 5'-ATC TGC CCT TCA GGA ACA GC-3'<br>R: 5'-AGC CTC CGA CTT GTG AAG TG-3'	59
LRAT	NM_022280.4	F: 5’-CAGGCTGAGAAGTTTCACGA-3’<br>R: 5’-CATCCACAAGCAGAACGGGA-3’	58
RPE65	NM_053562.3	F: 5′-GGAGACTATTAAGCAGGTTGATCT-3′<br>R: 5′-TCTTCCTTGTCTGCTTTCAGTG-3′	57
RLBP1	NM_001106274.1	F: 5′-CCACACTTTGCAGAAGGCTAAG-3′<br>R: 5′-AAGTTCACATAGCCTTTGAGCAGC-3′	58
RGR	NM_001107299.1	F: 5′-TATCCAGTCTCCTCCGGCGCTG-3′<br>R: 5′-GTTTCTGTCACCCCTAGAGTAGTCC-3′	62
RRH	NM_001107726.1	F: 5′-GATGTAACCAAGATGTCTGTGATGA-3′<br>R: 5′-AACATGGCCTTCCGAAACTTCTTA-3′	58
RDH5	XM_008765056.3	F: 5’- TGGAGCCTGGCTTCTTTC -3’<br>R: 5’- GTAGTGGGCCTGTATAGCTG -3’	55
RDH12	XM_032908769.1	F: 5’-GAGCTGGCCAAGCGGCTCC-3’<br>R: 5’-GAGGCGCCACAACAGGCACAT-3’	64
RDH13	NM_001108468.1	F: 5’- GCTGCCATGACCCTCATCAT -3’<br>R: 5’- CGTGGTCCAAACCAAAGCAG -3’	55
RDH14	NM_001109276.1	F: 5’- AAGAACTGCTACAGGAGGAGC -3’<br>R: 5’- CCCAGGTGGTTCACTCCAAA -3’	55
DRP1	XM_039087901.1	F: 5’-GACTTTGCTGATGCCTGTGG-3’<br>R: 5’-GTTGCCTGTTGTCGGTTCC-3’	58
NRF1	NM_001100708.1	F:5′-GCTGTCCCACTCGTGTCGTAT-3′<br>R:5′-GTTTGAGTCTAACCCATCTATCCG-3′	58
TFAM	NM_031326.1	F:5′-CGCCTAAAGAAGAAAGCACA-3′<br>R:5′-GCCCAACTTCAGCCATTT-3′	58
PGC1α	NM_031347.1	F: 5’-GCACACATCGCAATTCTCCC-3’<br>R: 5’-CTCTGCGGTATTCGTCCCTC-3’	59
HO1	NM_012580.2	F: 5'-TGCACATCCGTGCAGAGAAT-3'<br>R: 5'-CTGGGTTCTGCTTGTTTCGC-3'	55
SOD1	NM_017050.1	F: 5'-TTTTGCTCTCCCAGGTTCCG-3'<br>R: 5'-TGTCCTGACACCACAACTGG-3'	55
CAT	NM_012520.2	F: 5'-ACGAGATGGCACACTTTGACAG-3'<br>R: 5'-ACACCGGGGACCAAATGATG-3'	55
GPX1	NM_030826.4	F: 5'-CGGTTTCCCGTGCAATCAGT-3'<br>R: 5'-ACACCGGGGACCAAATGATG-3'	60

Table S2. Rat primer sequences using qRT-PCR (2)

Gene	Accession number	Sequence (5' - 3')	Tm (℃)
Angiogenin	XM_032917765.1	F: 5'-TCAGCACTATGATGCCAAGC-3'<br>R: 5'-GTGGTGATCTGGAAGGGAGA-3'	57
Endoglin	NM_001010968.3	F: 5'-AAG GTG TGA CTG TAC ACA AG-3'<br>R: 5'-CCA GAT CTG CAT ATT GTG GT-3'	56
PDGFRα	NM_012802.1	F: 5'-GAG GAC GAT TCT GCC ATC AT-3'<br>R: 5'-CAG TTC TGA CGT GGC TTT CA-3'	58
PDGFβ	NM_031524.1	F: 5'-CTC AGA GAG ATG GAG GTG CTC TC-3'<br>R: 5'-GCC CAG AGG AGT TCA TGT CTT AT-3'	60
βFGF	NM_019305.2	F: 5'-CCCGCACCCTATCCCTTCACAGC-3'<br>R: 5'-CACAACGACCAGCCTTCCACCCAAA-3'	66
FGF19	NM_130753.2	F: 5'-GAAATCTGTTGGAGTTCCGC-3'<br>R: 5'-AATCAGCCCGTATATCTTGC-3'	55
VEGF	NM_031836.3	F: 5'- ACGGACAGACAGACAGACAC -3'<br>R: 5'- CTTCTGGGCTCTTCTCTCTC -3'	55
PEDF	NM_177927	F: 5'- GATTGCCCAGCTGCCTTTGACA -3'<br>R : 5'-GGGACAGTCAGCACAGCTTGGATAG -3'	60
GAPDH	NM_017008.4	F: 5'- TCCCTCAAGATTGTCAGCAA -3'<br>R: 5'- AGATCCACAACGGATACATT -3'	58

Table S3. Human primer sequences using qRT-PCR

Gene	Accession number	Sequence (5’ - 3’)	Tm (℃)
HO-1	NM_002133.2	F:5′-TGGTGATGGCCTCCCTGTACCACATCT-3′<br>R:5′-AGAGCTGGATGTTGAGCAGGAACGCAGTCT-3′	60
SOD1	NM_000454.4	F:5′-GCTGTACCAGTGCAGGTCCTCA-3′<br>R:5′-CATTTCCACCTTTGCCCAAGTC-3′	60
RPE65	NM_000329.2	F: 5′-ATGGACTTGGCTTGAATCACTT-3′<br>R: 5′-GAACAGTCCATGAAAGGTGACA-3′	57
VEGFA	NM_001171623.2	F: 5’-GCCTTGCCTTGCTGCTCTAC-3’<br>R: 5’-ACATCCATGAACTTCACCACTTCG-3’	60
PEDF	NM_001329903.2	F: 5’- CCCATGATGTCGGACCCTAA -3’<br>R: 5’- TGTCATGAATGAACTCGGAGGTG -3’	55
GAPDH	NM_002046.7	F: 5’- GCACCGTCAAGGCTGAGAAC -3’<br>R: 5’- GTGGTGAAGACGCCAGTGGA -3’	60

Supplementary 1. Diabetic metabolism in STZ-induced rat model. The expression of body weight in STZ-induced rat model The expression of metabolic marker in STZ-induced rat serum by ELISA (A)ALT, (B)AST, (C)Albumin, (D)T-cholesterol, (E)TG, (F)HDL, (G)LDL, (H) BUN, (I)Creatinine, (J)BUN/Creatinine. P<0.05 The groups of experiments were as follow: Control, STZ: STZ-induced, Naïve : intravitreal transplantation of naïve PD-MSCs (STZ+ Naïve), and PD-MSCs[PEDF] (STZ + PEDF+).

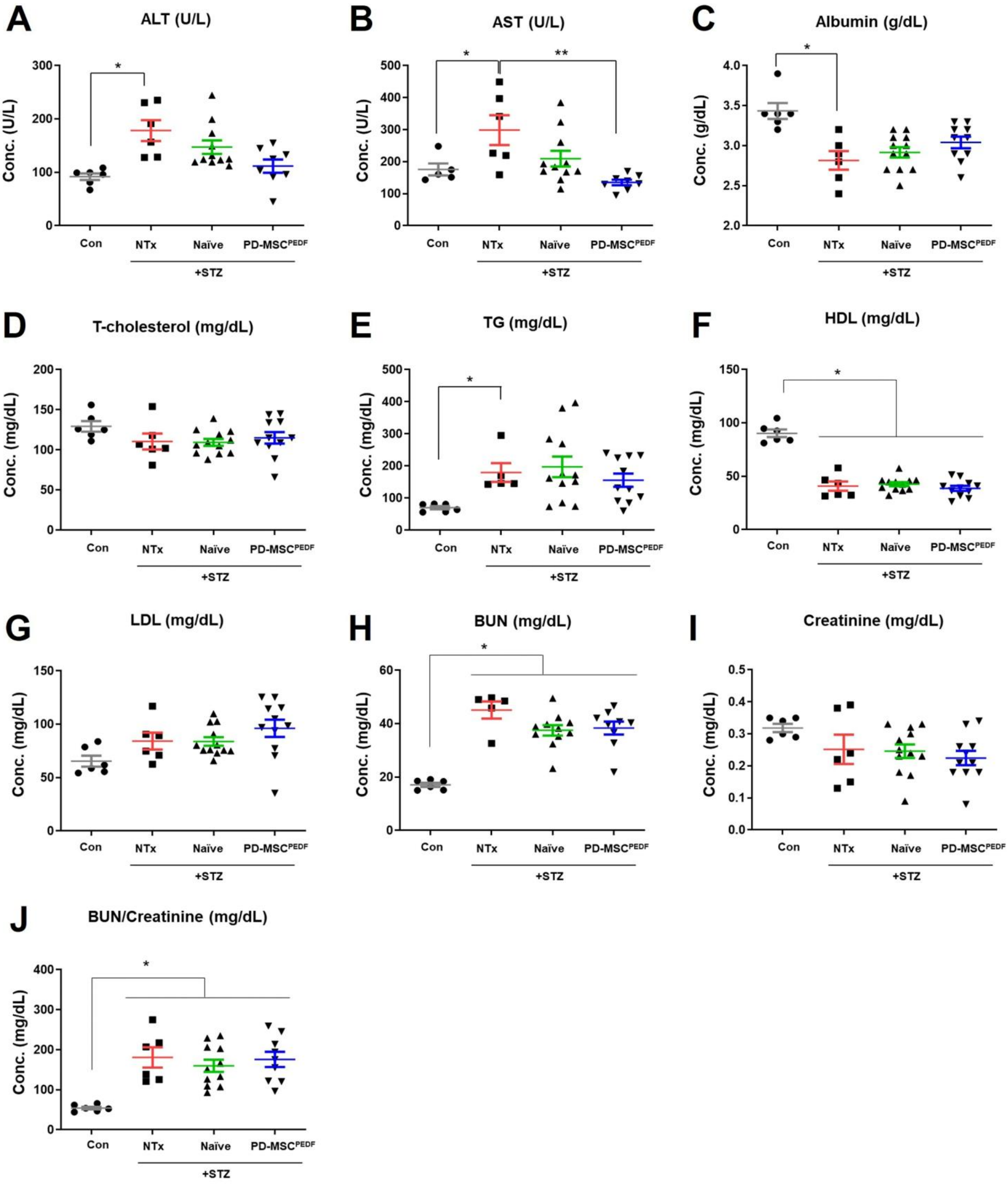

Supplement: Supplementary file 1 [file antioxidants-15-00473-s001.zip › antioxidants-4214369-supplementary.pdf]
